# Mid-Term Electrical Remodeling after Percutaneous Atrial Septal Defect Closure with GCO Device in a Pediatric Population

**DOI:** 10.3390/jcm12196334

**Published:** 2023-10-02

**Authors:** Jennifer Fumanelli, Silvia Garibaldi, Biagio Castaldi, Angela Di Candia, Alessandra Pizzuto, Domenico Sirico, Magdalena Cuman, Gianluca Mirizzi, Pietro Marchese, Massimiliano Cantinotti, Marcello Piacenti, Nadia Assanta, Cecilia Viacava, Giovanni Di Salvo, Giuseppe Santoro

**Affiliations:** 1 Pediatric Cardiology Unit, Woman’s and Child’s Health Department, Padua University, 35122 Padova, Italy; 2 Fondazione Toscana Gabriele Monasterio per la Ricerca Medica e di Sanità Pubblica, Electrophysiology Division, 56124 Pisa, Italy; 3 Fondazione Toscana Gabriele Monasterio per la Ricerca Medica e di Sanità Pubblica, Pediatric Cardiology and GUCH Unit, Heart Hospital “G. Pasquinucci”, 54100 Massa, Italy; apizzuto@ftgm.it (A.P.);; 4 Fondazione G. Monasterio CNR-Regione Toscana, Pediatric Cardiology and Cardiac Surgery, 56124 Pisa, Italy

**Keywords:** atrial septal defect (ASD), GORE^®^ Cardioform ASD Occluder device, P wave dispersion, QTc dispersion, cardiac electrical remodeling, arrhythmias

## Abstract

Background and aim: The GORE^®^ CARDIOFORM (GCO) septal occluder is an atrial septal defect/patent foramen ovale closure device with theoretical advantages over other commercialized devices thanks to its softness and anatomical compliance. Our aim was to evaluate the short- and medium-term electrocardiographic changes after percutaneous ASD closure with GCO in a pediatric population. Methods: We enrolled 39 patients with isolated ASD submitted to trans-catheter closure from January 2020 to June 2021. ECG was performed before, at 24 h and 6 months after the procedure. P wave dispersion, QTc and QTc dispersion were calculated. ECG Holter was recorded at 6 months after implantation. Results: Patients’ age and body surface area (BSA) were 8.2 ± 4.2 years and 1.0 ± 0.3 m^2^ respectively. At the baseline, mean P wave dispersion was 40 ± 15 msec and decreased at 24 h (*p* < 0.002), without any further change at 6 months. At 24 h, PR conduction and QTc dispersion significantly improved (*p* = 0.018 and *p* < 0.02 respectively), while the absolute QTc value considerably improved after 6 months. During mid-term follow-up, QTc dispersion remained stable without a significant change in PR conduction. The baseline cardiac frequency was 88.6 ± 12.6 bpm, followed by a slight reduction at 24 h, with a further amelioration at 6 months after the procedure (87.3 ± 14.2, *p* = 0.9 and 81.0 ± 12.7, *p* = 0.009, respectively). After device deployment, two patients developed transient, self-limited junctional rhythm. One of them needed a short course of Flecainide for atrial ectopic tachycardia. No tachy/brady-arrhythmias were recorded at the 6-month follow-up. ASD closure resulted in a marked decrease in right heart volumes and diameters at 6 months after percutaneous closure. Conclusions: Percutaneous ASD closure with the GCO device results in significant, sudden improvement of intra-atrial, atrio-ventricular and intraventricular electrical homogeneity. This benefit persists unaltered over a medium-term follow-up. These electrical changes are associated with a documented positive right heart volumetric remodeling at mid-term follow-up.

## 1. Introduction

Ostium secundum atrial septal defect (ASD) is a common congenital heart defect, found in about 1/1000 live births and leads to right chamber volume overload, pulmonary hypertension, systemic embolism, atrial arrhythmias and premature death over a long-term follow-up [1,2,3,4]. Atrial arrhythmias are well-known long-term complications of ASD [5], possibly due to chronic right atrial volume overload and resulting stretch [6,7].

In addition, also in other congenital and acquired heart diseases, P wave and QTc dispersion are well-known predictors of arrhythmias and are often considered as prognostic factors of morbidity and mortality.

The arrhythmic predictive role of P wave anomalies, that is, P wave duration and its dispersion on a 12-lead ECG, has also been discussed as the crucial determinant in a variety of clinical conditions, especially in paroxysmal atrial fibrillation (PAF) in healthy hearts. [8,9]. The prolongation of intra- and inter-atrial conduction times is the most predictive marker for idiopathic AF.

As the P wave conduction time is correlated to atrial arrhythmias, the prolongation of QTc dispersion is connected to the differences in recovery times in the various regional segments of the ventricular myocardium.

This correspondence is well associated with the increased risk of ventricular arrhythmias and sudden cardiac death for patients with coronary artery disease, chronic heart failure, diabetes mellitus or PAH [10,11,12].

The QT dispersion is also an indicator of electrical remodeling after ASD percutaneous closure, which is then linked to positive ventricular geometric change, and consequently, fewer arrhythmic complications [13,14].

Thus, decrease in P wave and QTc dispersion is supposed to assume a protective role in the development of atrial and/or ventricular arrhythmias [13,14,15,16,17]. Previous studies showed positive atrial and ventricular electrical remodeling after percutaneous ASD closure in terms of reduction of P wave and QTc dispersion, although the time-course of these changes may be partly hindered by the presence of the occluding device [14].

The new GORE^®^ Cardioform ASD Occluder device (WL Gore & Associates, Flagstaff, AZ, USA) (GCO) has been recently claimed as a significant technical innovation in transcatheter ASD treatment since it combines high softness and anatomic compliance with the potential to close large defects, even in challenging settings [18,19,20]. Thus, this device is an appealing alternative to other commercially available devices since it could supposedly have less impact on cardiac electrical remodeling, mainly in growing hearts.

This multi-center study aims for the first time to investigate the electrocardiographic changes after implantation of this novel device in pediatric and young adult patients.

## 2. Materials and Methods

### 2.1. Study Design and Population

This was a prospective single-arm study performed at the Pediatric Cardiology and GUCH Unit of the Heart Hospital “G. Pasquinucci” of Massa and the Pediatric Cardiology Unit of the University of Padua. The study was approved by our institutional review board, and informed consent was obtained from all patients involved in the study or their parents/guardians. We enrolled pediatric patients (age 5–18 years) with isolated, hemodynamically significant ASD submitted for percutaneous closure between January 2020 and June 2020. The following patients were excluded from the study: (i) patients with sinus venosus or primum type ASD, (ii) patients with inadequate rims, (iii) patients with significant cardiac/extra-cardiac comorbidities. A 12-lead surface ECG was recorded the day before the interventional catheterization, and at 24 h and 6 months after the procedure. In addition, we performed ECG-Holter monitoring at 1 and 6 months after device implantation in order to detect both cardiac rhythm changes and possible brady-or tachyarrhythmias.

Patients’ baseline characteristics, clinical history and medication were collected from medical records.

### 2.2. ASD Closure

**Figure 1:**
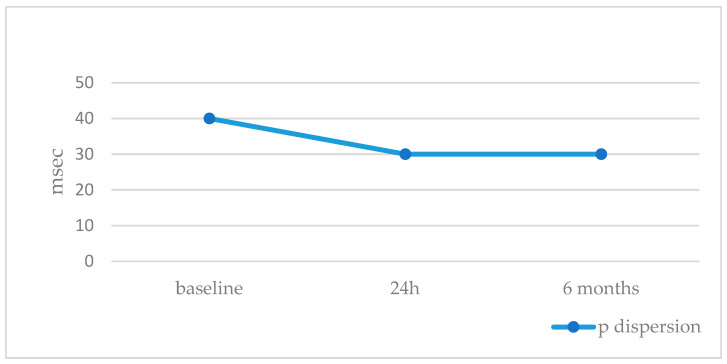
P dispersion trend during follow-up.

The interventional procedure was performed under general anesthesia by fluoroscopic with trans-esophageal echocardiographic (TEE) guidance. ASD size was assessed by static or dynamic sizing, based on the interventional cardiologist preference. Static sizing was performed by using the Amplatzer Sizing Balloon (Amplatzer Sizing Balloon, Abbott, Plymouth, MN, USA), which was inflated up to disappearance of the atrial shunt on TEE evaluation (“stop-flow” diameter). Dynamic sizing was accomplished by the pulling technique (“stretched” diameter) using an off-label sizing balloon (Equalizer Occluding Balloon, Meditech, Boston Scientific, Cork, Ireland). Afterwards, the size of the GORE Cardioform ASD occluder device was chosen based on the company indications [21,22,23]. The patients’ baseline characteristics and hemodynamic findings are summarized in Figure 1.

### 2.3. Electrocardiographic Findings

ECG analysis was blind on the status of the patients and manually performed by one experienced physician. A standard 12-lead electrocardiogram (ECG) was recorded at a rate of 25 mm/s and a calibration of 1 mV/cm for all the patients at the baseline and during follow-up. The P wave, PR interval, QRS amplitude, and QRS and QT dispersion were measured as follows in the C2 or V5 lead:

*P wave*: defined as the interval between the onset (junction of the isoelectric line at the beginning of the P wave deflection) to the offset (junction between the end of the P wave and the isoelectric line) of the P wave.

*P wave dispersion* (P dis = P max − P min) was calculated using these values.

*PR interval:* defined as the interval between the beginning of the P wave and the beginning of the QRS complex.

*QRS voltage:* defined as the amplitude measured from the nadir of the QRS complex to its peak.

*QRS duration:* defined as the maximum QRS duration in any lead from the first to the last sharp vector crossing the isoelectric line.

*QT interval:* defined as the interval between the beginning of the QRS complex and the end of the T wave. *QT dispersion* was defined as the difference between the maximum and the minimum of the QT intervals. The method for the corrected QT (QTc) interval measurement was performed by using the Bazett’s equation in any of the 12 ECG leads, but preferably in the C2 or V5 lead.

### 2.4. Echocardiographic Findings

We performed echocardiographic studies before and 6 months after the percutaneous procedure. We investigated both atrial and ventricular volumes, right and left ventricular diameters, their wall thickness and their function according to the Transthoracic Echocardiography Guidelines ACC/AHA/ASE 2019 [24].

### 2.5. Statistical Analysis

Statistical analysis was performed using SPSS for Windows v. 27 (SPSS, Chicago, IL, USA). Continuous variables were expressed as either mean ± SD or interquartile ranges when appropriate. The normal distribution was verified by using the Kolmogorov–Smirnov test. Categorical variables were presented as either absolute number or percentages. Post hoc analysis was performed with the Least Significant Difference (LSD) test. Student’s *t*-test correlation was used to evaluate the relationships between continuous variables.

The inter-operator and intra-operator variability and the Intra-Class Correlation Coefficient (ICC) were used to evaluate the intra- and inter-observer correlation.

Statistical significance was defined as *p* value < 0.05.

## 3. Results

**Figure 2:**
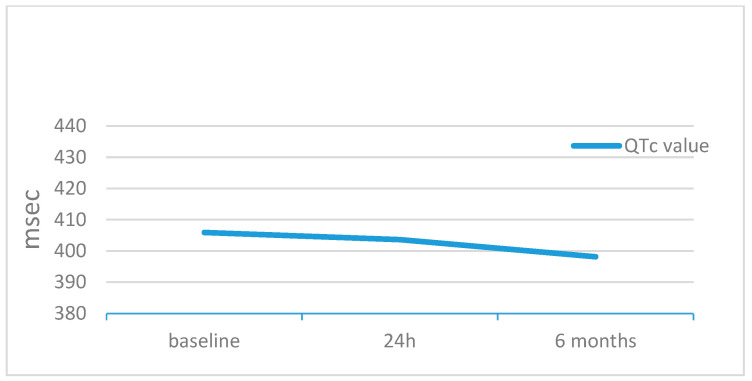
QTc trend during follow-up.

**Figure 3:**
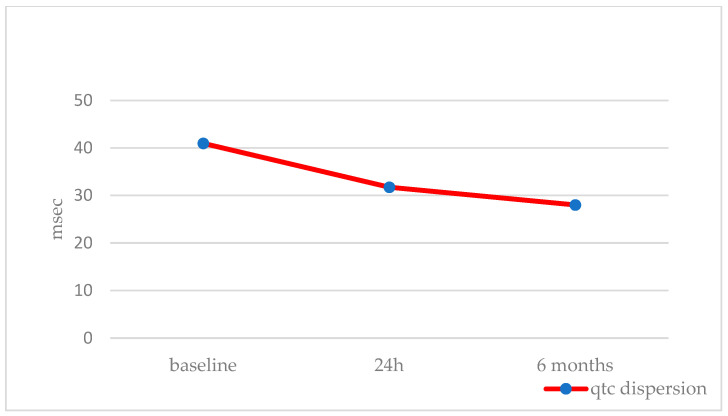
QTc dispersion trend during follow-up.

Overall, we included 39 patients. Patients’ ages and BSAs were 8.2 ± 4.2 years (IQR 4.2–8.3, median 7.0) and 1.0 ± 0.3 m^2^ (IQR 0.7–1.7, median 0.9), respectively. The stretched ASD diameter was 16.3 ± 4.5 mm (median 16), resulting in QP/QS of 1.7 ± 0.6 (median 1.5) (Table 1). The GCO device was successfully implanted in all patients. At the baseline ECG, cardiac rhythm was sinus in all patients, but one case showed low atrial rhythm. The baseline cardiac frequency was 88.6 ± 12.6 bpm. This value slightly decreased 24 h after the procedure (87.3 ± 14.2, *p* = 0.9), with a further significant decrease at 6 months (81.0 ± 12.7, *p* = 0,009) (Table 2). The baseline mean P wave dispersion was 40 ± 15 msec and decreased to 30 ± 13 msec (*p* < 0.002) at 24 h from the procedure, without any further change at 6 months (30 ± 13 msec, *p* < 0.002) (Figure 1, Table 2). PR conduction time significantly improved at 24 h (from 175.0 ± 20.8 to 144.0 ± 22.7 msec, *p* = 0.018) and did not significantly change at 6 months (164.0 ± 19.5 msec, *p* = NS) (Table 2). Ventricular electrical changes showed the same trend. Absolute QTc value did not significantly change at 24 h (from 405.9 ± 19.9 to 403.6 ± 17.3 ms, *p* = 0.5), but significantly improved at 6 months from the procedure (398.2 ± 15.5 msec, *p* = 0.03) (Figure 2, Table 2). However, QTc dispersion significantly decreased at 24 h (from 40.9 ± 13.3 to 31.7 ± 20.3, *p* < 0.02) and at 6 months (28.0 ± 18.1, *p* < 0.002) from the procedure (Figure 3, Table 2). After device deployment, two patients (5%) developed transient, self-limited junctional rhythm, and one of them needed a short course of anti-arrhythmic therapy (Flecainide) to prevent further supra-ventricular arrhythmias. Neither tachy- nor brady-arrhythmias or atrio-ventricular conduction abnormalities were recorded at the 6-month follow-up with ECG Holter monitoring.

According to the echocardiographic parameters, ASD closure resulted in a marked right atrial volume decrease (from 27.4 ± 7.9 to 17.2 ± 5.0, *p* < 0.0001) at 6 months after percutaneous closure associated with a contemporary increase in left atrial volume (from 17.1 ± 4.7 to 19.2 ± 5.8, *p* = 0,007) and left ventricular diameter (from 34.8 ± 8.0 to 40.5 ± 5.6, *p* < 0.0001). The right ventricular size reduced after six months (from 27.4 ± 5.1 to 18.3 ± 3.3, *p* < 0.0001) and its longitudinal systolic function normalized as well (24.4 ± 4.4 to 22.5 ± 4.5).

All the echocardiographic data are available at Table 3.

The inter-operator and intra-operator variability were, respectively, 5% and 4.2% for ECG parameters and less than 5% for echocardiographic data.

ICCs for intra-observer reproducibility showed a satisfactory correlation among repeated electrocardiographic and echocardiographic measurements (between 0.87 and 0.91). ICCs were between 0.73 and 0.88 about the inter-observer reproducibility.

## 4. Discussion

Supra-ventricular arrhythmias affect about 25% of ASD patients of adult age [5], presumably due to chronic right volume overload that results in atrial and ventricular stretch [7,25].

Over time, several ECG parameters have been proposed as markers of atrial and ventricular electrical vulnerability in ASD patients. Specifically, an increase in P wave dispersion has been correlated to an intra-atrial conduction delay and is potentially predictive of succeeding atrial arrhythmias [26,27]. The intra- and inter-atrial conduction time impairments have been deemed crucial determinants in a variety of clinical conditions, even in children with congenital heart disease, beyond the adult paroxysmal atrial fibrillation and pulmonary arterial hypertension [8,9,28]. Dilaveris et al. proposed the use of the P maximum duration and P wave dispersion as simple electrocardiographic predictive markers for the development of idiopathic paroxysmal atrial fibrillation [8]. In addition, Aytemir et al. found a significantly longer P maximum duration and greater P wave dispersion in patients affected by PAF [9].

A greater dispersion of the QTc is an important electrophysiological marker that is directly related to greater susceptibility to ventricular arrhythmias and, no less, to sudden cardiac death in cases of several comorbidities [10,11,12,13,14,15,16,17,18,19,20,21,22,23,24,25,26,27,28].

We know from the literature that the RV volume overload may also affect the QTc interval prolongation/dispersion in ASD patients [29,30]. However, these QTc anomalies were found in other congenital heart diseases (CHD), such as ventricular septal defect, patent ductus arteriosus, tetralogy of Fallot, atrioventricular septal defect and other complex congenital heart diseases. Thus, these changes may predispose patients to ventricular arrhythmias and potentially sudden cardiac death [31,32].

Accordingly, the change in P wave and QTc dispersion could be an important marker for arrhythmic events, strictly correlated to the potential atrial and ventricular electrical post-procedural remodeling.

Despite percutaneous ASD closure having fewer arrhythmic complications in the long-term follow-up compared with surgery [20], previous studies reported a transient worsening in P wave dispersion after device implantation. Santoro et al. [14] showed a significant post-procedural decrease in P wave dispersion in a small series of adult patients 6 months after percutaneous ASD closure with the Amplatzer Septal Occluder (Abbott, Plymouth MN, USA) device. However, P wave dispersion had slightly worsened 1 month after device implantation, and this transient change had been deemed as a “foreign body effect” of the occluding device. In support of this hypothesis, a previous paper by the same authors reported a similar positive effect on QT dispersion already at 1 month from percutaneous ASD closure, presumably as a result of a “pure” electrical remodeling not affected by the “mass effect” of the prosthesis on the ventricular chamber [13].

However, there are scant and conflicting data regarding the short-term atrial electrical remodeling after percutaneous closure of ASD in the pediatric population. Ozylimaz et al. [32] did not show any statistically significant change in P wave maximum duration and P wave dispersion at 24 h after percutaneous closure in a pediatric population. Conversely, Roushdy et al. [15] describes a positive electrical remodeling, in terms of reduction of P wave and QTc dispersion, at 24 h from ASD closure with the Amplatzer device, and these changes were confirmed at 6 months. However, no data concerning the P wave dispersion were reported in this study. Finally, Kaya et al. [13] showed a significant reduction in P minimum, P maximum, P dispersion, QRS duration and QT duration over a follow up period of 2 years in a large series of adults and children after ASD device closure.

The efficacy and safety profile of the GORE^®^ Cardioform Occluder device were recently confirmed by the post-marketing results [21,22,23]. Recently, Santoro et al. demonstrated a greater adaptability of this device to different and complex anatomies [20].

This current study demonstrated for the first time a statistically significant reduction in the P wave and QTc dispersion already at 24 h after ASD closure with the GCO device. These favorable changes remained stable during a mid-term follow-up. This improvement of the electrical intracardiac “milieu” could significantly reduce the arrhythmic risk of these patients and potentially prevent the arrhythmic burden recorded early after device implantation [11,12,26,31,32,33,34,35,36,37,38]. This positive electrical remodeling and the decreased arrhythmic complications could also be supported by the concurrent atrial and ventricular geometry changes. Specifically, the time course of the normalization of the volumetric parameters (Table 3) six months after closure of the defect appears to be synchronous on the electrocardiographic modifications.

This is the first time ever that a temporal correlation between the electrical and mechanical remodeling has been described in percutaneous ASD closure with GCO device. This result has been also confirmed unaltered during the mid-term follow-up.

In addition, we demonstrated that there is not a statistically significant correlation between the size of the ASD, the size of the device and the BSA of patients with P wave dispersion.

Finally, the trend of heart rate (HR) showed a significant decrease of the mean HR at 6 months after procedure. We could speculate that this parameter is related to the positive hemodynamic remodeling, without forgetting that external factors (such as childhood irritability) may falsify the absolute value at the moment of registration. In any case, these HR variabilities do not affect the trend of P wave and QTc dispersion, thanks to the application of Bazett’s equation and consequently the nullification of dependence on the cardiac frequency. We could therefore consider the latter parameter as an independent variable.

In our series, only one of the patients showed arrhythmias of intracardiac conduction abnormalities during a mid-term follow-up. One patient showed a burst of focal atrial tachycardia early after device implantation that was effectively treated with a short course of Flecainide.

This complication could have been due to both the large size of the device and the shortness of the surrounding rims.

## 5. Conclusions

Percutaneous closure of hemodynamically significant ASDs with the GORE^®^ Cardioform ASD Occluder in children results in significant, sudden improvement of intra-atrial, atrio-ventricular and intra-ventricular electrical homogeneity These favorable electrical changes persist unaltered over a mid-term follow-up, associated with a documented positive heart volumetric remodeling not hindered by the mechanical impact of the occluding device, and could explain the low rate of cardiac arrhythmias found at the mid-term ambulatory ECG evaluation in this series.

These post-procedural electrocardiographic results could therefore be considered as potential positive predictors of good electrical outcome even in the possible long-term follow-up. However, further studies are necessary to confirm potential advantages of this novel, softer device in decreasing the electrical risk of percutaneous ASD closure.

### Study Limitations

This study, aimed to evaluate the short- and mid-term electrical remodeling after ASD closure with this novel device in pediatric patients, has several limitations. Firstly, the sample size is very small, precluding any generalization of these results. However, the clinical and hemodynamic characteristics of these patients were very homogeneous. Indeed, all patients showed large, hemodynamically significant ASD that, in most cases, made them potential candidates for surgical repair. Secondly, no comparison with other commercially available devices, in particular, the most frequently used Amplatzer device, was performed. However, almost all of the scant data reported in the literature were obtained from patients treated with this device, making this comparison unnecessary and redundant, in our view. Although the inter-observer variability was acceptable, manual measurements of ECG parameters remain difficult at 25 mm/s paper speed. Therefore, this setting parameter could represent another study limitation. Further studies are necessary to confirm if increased paper speed or automated interpretation could improve the reliability of data.

## Figures and Tables

**Table 1:** Baseline characteristics.

Baseline Characteristics	Findings
Median age (yrs)	8.2 ± 4.2 (median 7)
Mean of BSA (m^2^)	1.0 ± 0.3 (median 0.9)
ASD**stretched diameter (mm)	16.3 ± 4.5
Balloon sizing (yes/no)	39 (all)
QP/QS	1.7 ± 0.6 (median 1.5)

**Table 2:** Trend of electrocardiographic parameters during follow-up.

	Baseline	24 h	6 Months	*p* (24 h)	*p* (6 Mo)
HR [bpm]	88.6 ± 12.6	87.3 ± 14.2	81.0 ± 12.7	*p* = 0.9	*p* = 0.009
P Wave Dispersion [ms]	40 ± 15	30 ± 13	30 ± 13	*p* < 0.002	*p* < 0.002
PR Conduction [ms]	175.0 ± 20.8	144.0 ± 22.7	164.0 ± 19.5	*p* = 0.018	N.S.
QTc Dispersion [ms]	40.9 ± 13.3	31.7 ± 20.3	28.0 ± 18.1	*p* < 0.02	*p* < 0.002
QTc value [ms]	405.9 ± 19.95	403.6 ± 17.28	398.2 ± 15.5	*p* = 0.5	*p* = 0.03

**Table 3:** Trend of echocardiographic parameters at the baseline and after 6 months.

	Baseline	6 Months	*p*-Value
**IVSd (mm)**	6.5 ± 1.2	6.6 ± 1.2	
**LVd (mm)**	34.8 ± 8.0	40.5 ± 5.6	<0.0001
**LVPWd (mm)**	6.1 ± 1.1	6.4 ± 1.2	
**LVs (mm)**	22.3 ± 3.7	25.6 ± 4.6	0.0001
**LVEF (%)**	67.3 ± 6.2	67.5 ± 5.6	
**RVd (mm)**	27.4 ± 5.1	18.3 ± 3.3	<0.0001
**RVd/LVd ratio**	0.78 ± 0.09	0.45 ± 0.7	<0.0001
**LAVI (ml/mq)**	17.1 ± 4.7	19.2 ± 5.8	0.007
**RAVI (ml/mq)**	27.4 ± 7.9	17.2 ± 5.0	<0.0001
**E/A**	1.7 ± 0.4	1.9 ± 0.5	0.0017
**E/E’**	6.2 ± 1.7	6.6 ± 2.4	
**TAPSE (mm)**	24.4 ± 4.4	22.5 ± 4.5	

**Legend:** interventricular septum (IVS), left ventricle (LV), left ventricle posterior wall (LVPW), left ventricle ejection fraction (LVEF), right ventricle (RV), left atrial volume index (LAVI), right atrial volume index (RAVI), diastole(d), systole (s), tricuspid annular plan systolic excursion (TAPSE).

## Data Availability

Data were derived from the management system of patients at the Children’s Hospital, Pediatric Cardiology Unit, of the Padua and Pediatric Cardiology Division of Fondazione Monastiero.
